# From phagocytosis to metaforosis: Calcineurin’s deadly role in innate processing of fungi

**DOI:** 10.1371/journal.ppat.1006627

**Published:** 2018-01-04

**Authors:** Darius Armstrong-James, Leon de Boer, Amelia Bercusson, Anand Shah

**Affiliations:** Fungal Pathogens Laboratory, National Heart and Lung Institute, Imperial College London, London, United Kingdom; McGill University, CANADA

## Susceptibility to fungal disease in organ transplantation

Opportunistic fungal infections are a major complication of organ transplantation, with a yearly incidence of 3.1% of invasive fungal disease in the first 3 years after transplantation in large prospective studies [1]. Amongst transplant recipients, lung transplantation recipients are at very high risk of pulmonary aspergillosis, a fatal chronic and progressive lung infection due to the mold *Aspergillus fumigatus* [1]. Additionally, colonization with *A*. *fumigatus* is a key risk factor for the development of bronchiolitis obliterans, leading to organ rejection [2]. While the role of steroids in susceptibility to fungal disease in transplant populations is well appreciated, our understanding of how calcineurin inhibitors, now the core backbone of transplant immunosuppression, affect immunity to fungi has not been extensively studied until recently.

The two calcineurin inhibitors widely used in clinical practice, cyclosporin and tacrolimus (FK506), are both thought to inhibit organ cellular rejection responses though inhibition of T-cell lymphoproliferative responses to donor antigen presentation. However, emerging data have demonstrated a wider role for calcineurin across a range of cell types and tissues [3]. Calcineurin–nuclear factor of activated T cells (NFAT) signaling plays an important role in cardiac development, neurological syndromes, and osteoclast differentiation [4]. Notably, an important role of calcineurin in innate immunity has emerged, with calcineurin-dependent innate immune responses important for immunity to *Candida albicans*, *A*. *fumigatus*, and *Eschereschia coli* [5]. Taken together, these observations suggest that a better understanding of the impact of calcineurin inhibitors on innate immune responses to opportunistic pathogens is required.

## Calcineurin’s central role in calcium-dependent cellular responses via NFAT

Calcineurin has long been recognized as a fundamental calmodulin-dependent serine-threonine phosphatase that enables integration of a range of cellular signals that result in calcium flux. Subsequent activation of calcineurin phosphatase leads to dephosphorylation of the NFAT family of transcriptions factors, which are imported into the nucleus to direct transcriptional responses [6]. It is postulated that the NFAT family arose in vertebrates some 500 million years ago as a consequence of a recombination event between the Rel homology domain of nuclear factor kappa-light-chance-enhancer of activated B cells (NF-κB) and a calcium-responsive element analogous to the Calcium responsive zinc finger 1 (Crz1) zinc family transcription factor in fungi. This resulted in a hybrid transcription factor that hardwires calcium flux to immune control, allowing coordinate evolution of vertebrate organogenesis and adaptive immune function [6]. In Mammalia, there are 5 NFAT isoforms, of which NFAT 1–4 are variably expressed in myeloid and lymphoid compartments. These different isoforms enable a diversification of the dynamic transcriptional response that may occur in response to the kinetics of calcium transients, which may allow fine-tuning of calcium-responsive signaling appropriate to the cogent effector requirement of the individual cell type and stimulus [7]. Given the physiological relationship between calcium flux and cytoskeletal remodeling, in the context of pathogen internalization, this suggests that the calcineurin pathway may be able to tailor systems-level immune responses to cellular uptake or invasion by microbes in a manner that can discriminate physical characteristics of the invading pathogen such as size, shape, or movement.

## NFAT-independent interactions enable diversification of calcineurin function

While most focus has been concentrated on understanding the role of calcineurin in transcriptional responses via NFAT, calcineurin has a wide range of substrates as well as other protein interactions. Calcineurin is a heterodimer consisting of a catalytic A subunit and a regulatory B subunit. Calcineurin A binds calmodulin in a Ca^2+^-dependent manner, leading to calcineurin activation [6]. Calcineurin then dephosphorylates NFAT, leading to nuclear shuttling. Calcineurin has also been shown to directly interact with TWICK-related spinal cord K^+^ channel (TRESK), which is important in nociception [8]; with Kinase Suppressor of RAS (KSR2), a protein scaffold that enables signaling from RAS to Extra-cellular signal-Regulated Kinase (ERK) and subsequent ERK activation [9]; as well as with A Kinase Anchoring Protein 79 (AKAP79; an anchoring molecular enabling colocalization of calcineurin with protein kinase A [PKA] and protein kinase C [PKC]) [10, 11] and the regulator of calcineurin 1 (RCAN1) family of endogenous calcineurin negative regulators (11). Interestingly, a recent phosphoproteomic screen in human macrophages further suggested that calcineurin further regulates the actin cytoskeleton, cell cycle, Mitogen-Activated Protein (MAP) kinase pathways, and angiogenesis [12]. A better understanding of these NFAT-independent pathways is now required to delineate the wider role that calcineurin plays in calcium-dependent cellular responses.

## The calcineurin–NFAT pathway is activated during phagocytosis

**Fig 1:**
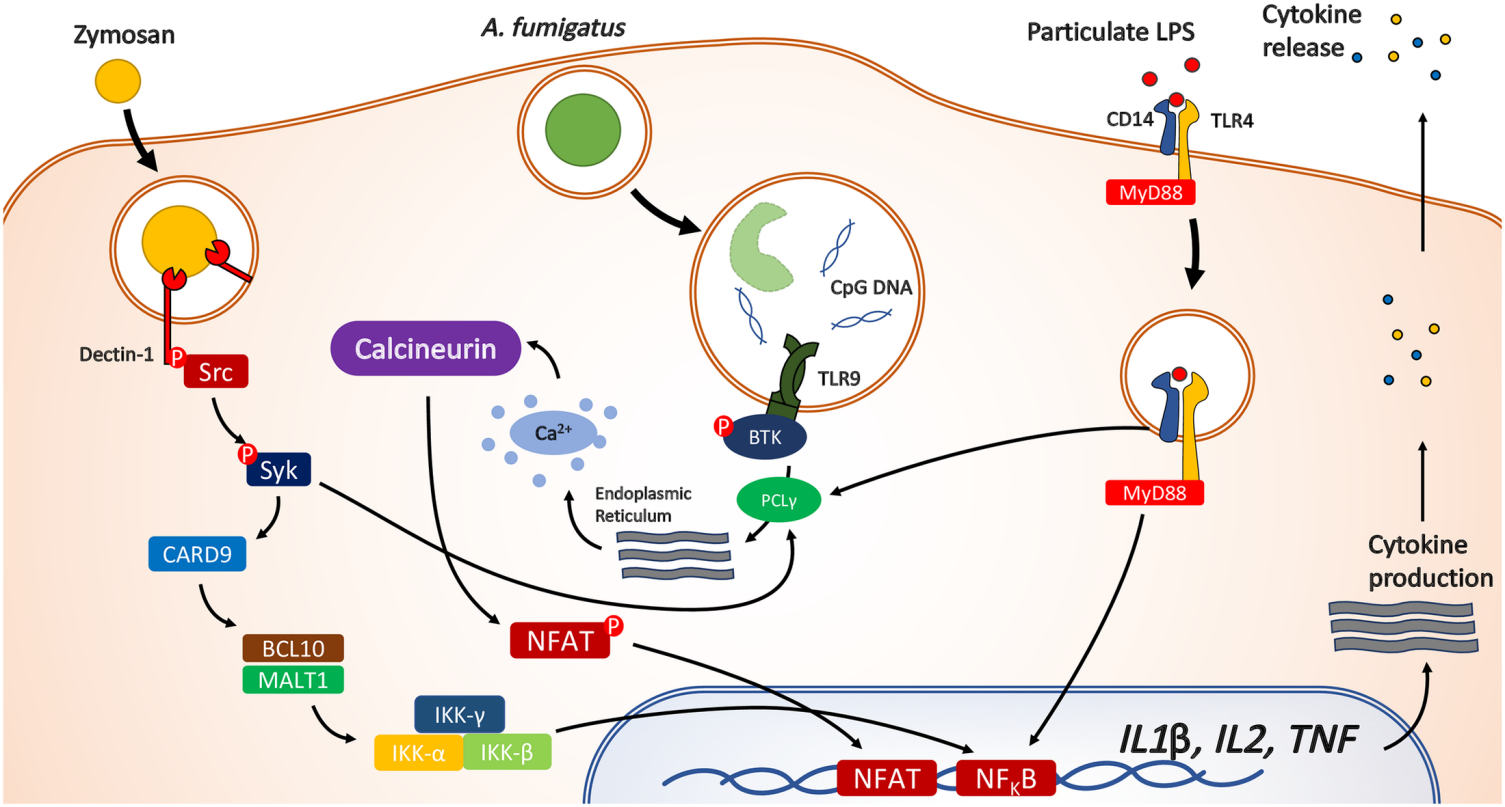
Pathways to calcineurin–NFAT activation during endocytosis. NFAT, nuclear factor of activated T cells. Phagocytosis of zymosan or *C. albicans* leads to Syk-dependent activation of calcium flux via PLCγ. In contrast, for *A. fumigatus*, phagocytosis leads to TKR9 and Btk-dependent calcineurin-NFAT activation via PLCγ. This is independent of Syk. For particulate LPS, endocytosis via CD14 leads to PLCγ-dependent calcineurin-NFAT activation.

The activation of the calcineurin–NFAT pathway has been shown to be the consequence of phagocytosis of a number of different particulate microbial-associated molecular patterns (5). For example, phagocytosis of particulate lipopolysaccharide (LPS) leads to CD14-dependent phospholipase C gamma 2 (PLCɣ2) activation, calcium flux, and subsequent calcineurin–NFAT signaling (Fig 1) [13]. Similarly, uptake of zymosan fungal particles leads to Dectin-1 and spleen tyrosine kinase (Syk)-dependent activation of calcineurin–NFAT signaling [14]. Our studies with the pulmonary fungal pathogen *A*. *fumigatus* further demonstrate that macrophage phagocytosis is essential for calcineurin–NFAT signaling, through a pathway that is dependent on both endosomal sensing via toll-like receptor (TLR) 9 as well as Bruton’s tyrosine kinase [15]. Interestingly, both phagocytosis of *A*. *fumigatus* and subsequent calcineurin–NFAT signaling were found to be independent of Syk and of myeloid differentiation primary response 88 (Myd88) [15]. RNAseq analysis of the human macrophage calcineurin-dependent response to *A*. *fumigatus* infection revealed regulatory control of caspase pathways, nucleotide-binding oligomerization domain-containing protein (NOD) pathways, interleukin 1 (IL-1) signaling, and the Activator Protein 1 (AP-1) transcriptional network, and calcineurin was found to regulate tumor necrosis factor (TNF)-α, granulocyte macrophage colony-stimulating factor (GM-CSF), and monocyte chemoattractant protein 1 (MCP-1) levels [12]. Calcineurin inhibition led to mild impairment of phagocytic capacity of human macrophages for *A*. *fumigatus* and increased early lysosomal fusion [12]. Interestingly, calcineurin has been shown to inhibit lysosmal fusion during mycobacterial infection [16]. Ultimately, effective airway calcineurin signaling was crucial for early recruitment of neutrophils at the site of infection by enabling optimal chemokine responses. Notably, understanding the role of Btk signaling for myeloid immunity to *A*. *fumigatus* has recently become of great importance [15] because clinically licensed Bruton’s tyrosine kinase (Btk) inhibitors have been found to have a strong association with risk for disseminated aspergillosis [17]. While Btk is known to mediate calcium flux via PLCγ2 recruitment to its Src homology 2 (SH2) domain, how it is activated by fungi or in innate immunity has not been characterized in detail. Furthermore, how the calcineurin pathway cooperates with the key signaling pathways for *Aspergillus* sensing via key recognition molecules such as Pentraxin 3, Dectin-1, Dectin-2, Dectin-3, and TLR4 has not been investigated [18]. However, a number of innate pattern recognition receptors involved in antifungal immunity contribute to calcium flux upstream of calcineurin activation, such as the TLRs, C-type lectins, and opsonic receptors, likely convergent on PLC-γ2 activation, resulting in the generation of inositol 1,4,5-trisphosphate and the release of calcium ions from intracellular stores into the cytoplasm [19]. In dendritic cells, engagement of CD14 and TLR4 by LPS leads to activation of Src-family kinase and PLC-γ2 [13]. In contrast, fungal zymosan binds Dectin-1, enabling Syk docking and subsequent PLC-γ2 activation [14]. Opsonic phagocytosis through Fragment crystallizable gamma Receptor (FcγR), which is also important for fungal phagocytosis, may also drive calcium flux via Syk [20]. Notably, calcium flux plays an important role in mast cell, eosinophil, and natural killer cell responses; however, the relevance to fungal immunity is less clear [19].

Subsequent analysis of the role of calcineurin in human myeloid cells revealed that calcineurin inhibits the proliferation of myeloid granulocyte-monocyte progenitors via FMS-like Tyrosine Kinase 3 Ligand (Flt3-L) [21]. Furthermore, calcineurin regulates CD103^+^ dendritic cell IL-2 production and subsequent control of T helper 17 (Th17) responses. Consistent with these observations, impaired calcineurin signaling in CD11c-positive myeloid cells leads to increased susceptibility to murine hematogenous aspergillosis [22]. In contrast, studies with the fungal pathogen *C*. *albicans* further reveal that calcineurin may play an important role in neutrophil-mediated sterilizing responses to hematogenous infection, in a Dectin-1 and Syk-dependent fashion, as well as *IL10*, *Cox2*, *Egr1*, and *Egr2* expression [23]. These observations indicate that calcineurin plays diverse roles in coordinating appropriate myeloid immune responses to different fungi.

## Calcineurin regulates cell death responses during fungal infection

**Fig 2:**
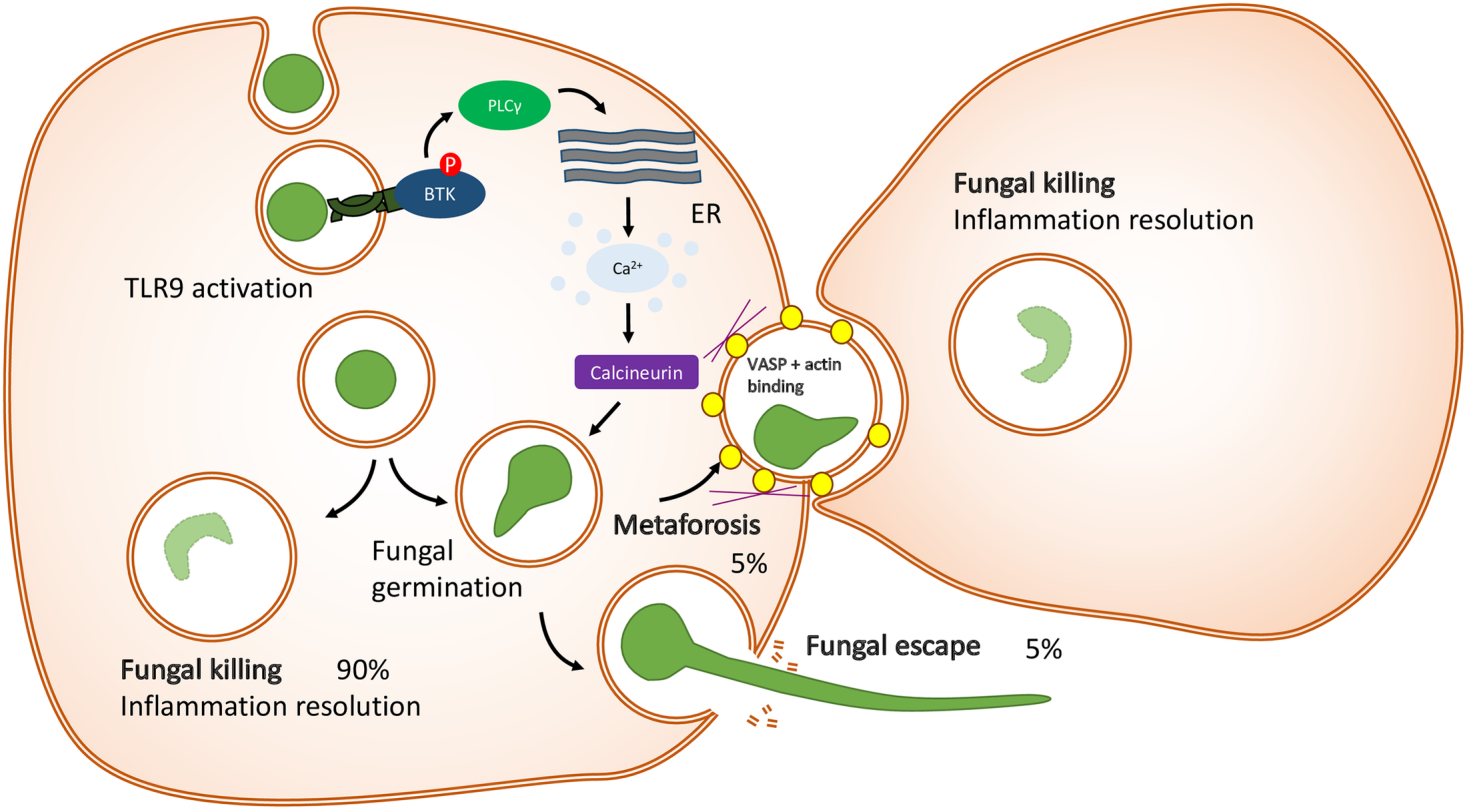
Metaforosis—lateral transfer of *A*. *fumigatus* during macrophage necrosis. For 90% of macrophages, phagocytosis of *A*. *fumigatus* leads to successful killing of the pathogen and resolution of the inflammatory response. However, if progressive fungal germination occurs, this results in inflammatory macrophage cell death. Under these circumstances, around half of germinating conidia ultimately escape from the dying macrophage. However, in the other half of cases, the dying macrophage is able to transfer the germinating conidium to a bystander macrophage, giving this macrophage a second chance to kill the fungus. This process of lateral transfer, or metaforosis, is highly dependent on calcineurin activation and occurs through a late endocytic compartment surrounded by actin and VASP. VASP, vasodilator-stimulated phosphoprotein.

Recently, we described the role of calcineurin in human macrophage responses to *A*. *fumigatus* [12]. Our studies indicated that calcineurin has a role in both phagocytic uptake of conidia as well as fungal killing. Real-time confocal microscopy revealed that calcineurin regulated macrophage-programmed necrosis responses that occurred specifically in macrophages in which successful fungal germination in the late phagosome was occurring (Fig 2). Unexpectedly, we observed that macrophage-programmed necrosis resulted in lateral transfer of germinating conidia to bystander live macrophages, in a process known as “metaforosis” (from the Greek “metofor,” to transfer). This enabled successful control of fungal germination in recipient macrophages, indicative of a process whereby cell–cell cooperation enables control of infection in the myeloid compartment. Subsequent phosphoproteomic analysis identified vasodilator-stimulated phosphoprotein (VASP) as a key actin polymerase regulated through calcineurin. Both VASP and actin were observed to colocalize to the late endosomal compartment through which metaforosis occurred during macrophage necrosis events.

Notably, calcineurin also regulated the cell cycle protein Cell Division Cycle 25A (CDC25A), required for progression from Gap 1 (G1) to the Synthesis (S) phase of the cell cycle. Interestingly, similar transfer events have previously been visualized but not characterized or related to cell death for *Cryptococcus neoformans* and happen at a much lower frequency [24]. Instead, vomocytosis—or extracellular expulsion of yeast—is seen more commonly for *C*. *neoformans*. This has been shown to be regulated by the atypical kinase Erk5 [25]. Further studies in granulocyte-monocyte progenitors show that calcineurin–NFAT regulates expression of the cell cycle genes cyclin-dependent kinase (Cdk) 4, Cdk6, and Cdkn1a, promoting cell cycle progression and cellular differentiation. These observations parallel long-established concepts in lymphocyte biology in which calcineurin inhibitors have an established clinical role as inhibitors of lymphoproliferative responses [26]. Thus, accumulating evidence places calcineurin as a central regulator of both cellular differentiation, proliferation, and cell death decisions in myeloid immunity.

## Conclusions and perspective

Calcineurin is the major target of immunosuppressive therapy in clinical transplantation, with the principal aim of inhibiting T-cell proliferative responses to donor organ antigens. However, invasive fungal diseases have emerged as a major complication of organ transplantation and are thus a barrier to aggressive immunosuppression strategies. Rapid progress over the last decade has redefined our understanding of the role of calcineurin as a major player in the innate immune response. In particular, we now understand that the calcineurin–NFAT pathway is triggered by phagocytosis of particulate ligands, bacteria, and eukaryotic pathogens such as fungi through endocytic recognition pathways. Subsequent activation of the calcineurin–NFAT pathway enables coordinated regulation of innate immune responses through efficient neutrophil chemotaxis to the site of infection, as well as dendritic cell–mediated activation of T-cell responses. An important role of calcineurin in myeloid differentiation and cell death decisions during fungal infection has recently emerged. Calcineurin has now been recognized to be a major regulator of “metaforosis,” a novel form of macrophage-programmed necrosis during which cell-to-cell transfer of germinating fungal conidia through a late endosomal compartment enables subsequent control of infection in recipient macrophages. Understanding the wider impact of calcineurin-dependent innate responses on inflammatory pathways during chronic fungal infection is now a key area for further study, which may enable further modulation of dysregulated immune responses (Table 1).

**Table 1:** Important future questions in the field.

What calcineurin-independent calcium signaling pathways are important for innate fungal immunity?
Is Bruton’s tyrosine kinase a primary driver of macrophage calcium flux in response to other fungi?
Does calcineurin contribute to macrophage cell death responses to other fungi apart from *A*. *fumigatus*?
Can modulation of calcineurin signaling improve outcomes from fungal disease?
How important is calcineurin signaling for innate immunity to other nonfungal pathogens?
How does calcineurin modulate macrophage-programmed cell death responses?
